# Long-term methyl jasmonate exposure triggers COI1-independent induction of myrosinases *TGG1* and *TGG2* in Arabidopsis

**DOI:** 10.3389/fpls.2026.1801473

**Published:** 2026-03-30

**Authors:** Mohamadreza Mirzaei, Andisheh Poormassalehgoo, Kaichiro Endo, Shino Goto-Yamada, Ewa Dubas, Kenji Yamada

**Affiliations:** 1 Malopolska Centre of Biotechnology, Jagiellonian University, Krakow, Poland; 2 Center for Advanced Technology, Adam Mickiewicz University, Poznan, Poland; 3 Doctoral School of Exact and Natural Sciences, Jagiellonian University, Krakow, Poland; 4 The Franciszek Górski Institute of Plant Physiology, Polish Academy of Sciences, Krakow, Poland

**Keywords:** chemical defense, COI1, FAMA, methyl jasmonate, myrosin cell, myrosinase

## Abstract

Plants are constantly subjected to stresses such as herbivore attacks, which continuously activate the jasmonic acid (JA) signaling pathway in nature. However, knowledge about the effect of long-term activation of the JA signaling pathway on plant defense response remains limited. THIOGLUCOSIDE GLUCOHYDROLASE 1 (TGG1) and TGG2 are enzymes that activate defensive metabolites, namely glucosinolates, which are involved in defense against herbivores and pathogens. Here, we show that prolonged exposure to the wounding hormone methyl jasmonate (MeJA) enhances *TGG1* and *TGG2* expression in the rosette leaves of 12-d-old *Arabidopsis thaliana*, and this response does not appear to be strictly dependent on the canonical JA signaling pathway. Airborne MeJA treatment for up to 5 d enhanced both *TGG1* and *TGG2* gene expression and their protein levels in Arabidopsis leaves. Notably, *TGG1* and *TGG2* gene expression was also significantly upregulated in two JA signaling pathway mutants, namely *coi1–16* and *myc2,3,4*, following 5 d of MeJA treatment. TGG1 and TGG2 proteins accumulate in specialized myrosin cells of rosette leaves. Myrosin cell area expanded in response to MeJA treatment in a leaf-age-dependent manner. Consistent with this observation, the expression of *FAMA*, a transcription factor known to regulate *TGG1* and *TGG2* expression, was also increased in a leaf-age-dependent manner after 5 d of MeJA treatment. Taken together, our results suggest the existence of additional regulatory mechanisms beyond canonical JA signaling pathway that is activated by long-term exposure to MeJA and regulates the expression of defense-related genes *TGG1* and *TGG2*.

## 1 Introduction

The myrosinase-glucosinolate system is a chemical defense mechanism against herbivores and pathogens in plants of the order Brassicales. This defense mechanism was first discovered in mustard seeds (Bussy, 1840; Bones and Rossiter, 1996) and consists of a family of enzymes called myrosinase and a class of substrates called glucosinolate. Myrosinases and glucosinolates are stored in different cells or subcellular compartments in plants (Shirakawa et al., 2016). However, these enzymes and substrates come into contact when tissue damage occurs, and the enzymes begin to hydrolyze glucosinolates to produce toxic compounds, such as isothiocyanate, to stop further damage by herbivores and pathogens (Bhat and Vyas, 2019). In *Arabidopsis thaliana*, canonical myrosinase activity is encoded by six *THIOGLUCOSIDE GLUCOHYDROLASE* (*TGG*) genes, namely *TGG1*–*TGG6* (Rask et al., 2000; Shirakawa and Hara-Nishimura, 2018). Two of the six genes, *TGG1* (*At5g26000*) and *TGG2* (*At5g25980*), are expressed in the leaves, whereas *TGG4* (*At1g47600*) and *TGG5* (*At1g51470*) are expressed in the roots (Shirakawa et al., 2016; Andersson et al., 2009). *TGG3* (*At5g48375*) and *TGG6* (*At1g51490*) were considered pseudogenes in Col-0 because of their open reading frame disruption (Wang et al., 2009).

In Arabidopsis, TGG1 and TGG2 proteins are stored in two types of cells with similar ‘idioblast’ characteristics: myrosin cells and stomata guard cells (Shirakawa et al., 2022). Myrosin cells are located next to phloem cells, and this particular name was given because myrosinases accumulate extensively in their vacuoles (Guignard, 1890; Heinricher, 1884; Bones and Rossiter, 1996). Although myrosin cells are localized adjacent to the phloem, they develop independently of vascular precursor cells and originate directly from ground meristem cells (Shirakawa et al., 2014, 2016). Basic helix-loop-helix (bHLH) transcription factors (TFs) FAMA, SCREAM1 and SCREAM2, which regulate stomatal guard cell differentiation, regulate the differentiation of myrosin cells (Li and Sack, 2014; Shirakawa et al., 2014). Although myrosinases have a chemical defense function in both myrosin and stomatal cells, it is reported that glucosinolate metabolism mediated by TGG1 in the stomatal cells plays a role in stomatal movement (Zhang et al., 2019).

Jasmonic acid (JA) and its metabolic derivatives, such as jasmonoyl-isoleucine (JA-Ile) and methyl jasmonate (MeJA), are cyclopentanone derivatives of linolenic acid (Devoto et al., 2002; Liu and Timko, 2021). These compounds are widely distributed phytohormones in higher plants and play important roles in the stress responses and regulation of various plant developmental processes (Ruan et al., 2019). In the canonical JA signaling pathway, the bioactive ligand JA-Ile is sensed by CORONATINE INSENSITIVE 1 (COI1)-JASMONATE ZIM-DOMAIN (JAZ) co-receptor complex (Xie et al., 1998; Devoto et al., 2002). COI1, a leucine-rich repeat (LRR)-F-box protein, forms the Skp1–Cul1–F-box-protein (SCF) ubiquitin ligase complex, which is called SCF^COI1^. Upon ligand binding, SCF^COI1^ promotes interaction with JAZ repressors, leading to their ubiquitination and their subsequent degradation by the 26S proteasome (Ruan et al., 2019). In the non-JA-signaling state, JAZ proteins physically interact with MYC2/3/4 TFs and repress their functions (Chini et al., 2009; Ruan et al., 2019). JA-induced degradation of JAZ proteins releases downstream TFs that activate various JA-inducible genes. In Arabidopsis, JA-inducible marker genes include *VEGETATIVE STORAGE PROTEIN 2* (*VSP2*), *JAZ2*, *JASMONATE RESPONSIVE 1* (*JR1*), and *BETA GLUCOSIDASE 18* (*BGLU18*) (Liu and Timko, 2021; Schweizer et al., 2013; Stefanik et al., 2020; Feng et al., 2021) (**Supplementary Figure 1**).

It has been reported that myrosinase activity is significantly lower in the *coi1* mutant than in the wild type when sinigrin is used as a substrate (Capella et al., 2001), and MeJA treatment increases *TGG1* expression in a COI1-dependent manner in Arabidopsis (Feng et al., 2021). Together, these findings support the prevailing model in which JA perception by the SCF^COI1^ complex and subsequent activation of MYC2/3/4 transcription factors are essential for jasmonate-induced expression of myrosinase genes. However, this model is largely derived from short-term jasmonate treatments conducted under controlled laboratory conditions. In contrast, plants in natural conditions frequently encounter prolonged stresses such as recurrent herbivore attacks and wounding, which continuously induce JA biosynthesis and long-lasting activation of the JA responses (McConn et al., 1997; Halitschke and Baldwin, 2004). Whether canonical COI1–MYC signaling module fully accounts for JA-regulated gene expression under such long-term stress conditions remains largely unexplored.

To address this gap, we investigated the JA responses of *TGG1* and *TGG2* under continuous long-term MeJA treatment (5 d exposure) in wild-type Arabidopsis and in mutants lacking key components of the canonical JA signaling pathway, namely, *coi1–16* and *myc2,3,4*. This experimental design allowed us to examine the JA-dependent regulation of myrosinase genes under conditions that more closely resemble sustained stress in nature. Unexpectedly, we found that although *TGG1* and *TGG2* are JA-responsive, their long-term induction by MeJA is not fully dependent on canonical JA signaling pathway components, such as COI1 and MYC2/3/4. These results uncover a previously unrecognized mode of JA-responsive gene regulation and suggest the existence of alternative or compensatory mechanisms that maintain myrosinase-related defense responses during prolonged MeJA treatment.

## 2 Materials and methods

### 2.1 Plant materials, plant growth condition

**Figure 1:**
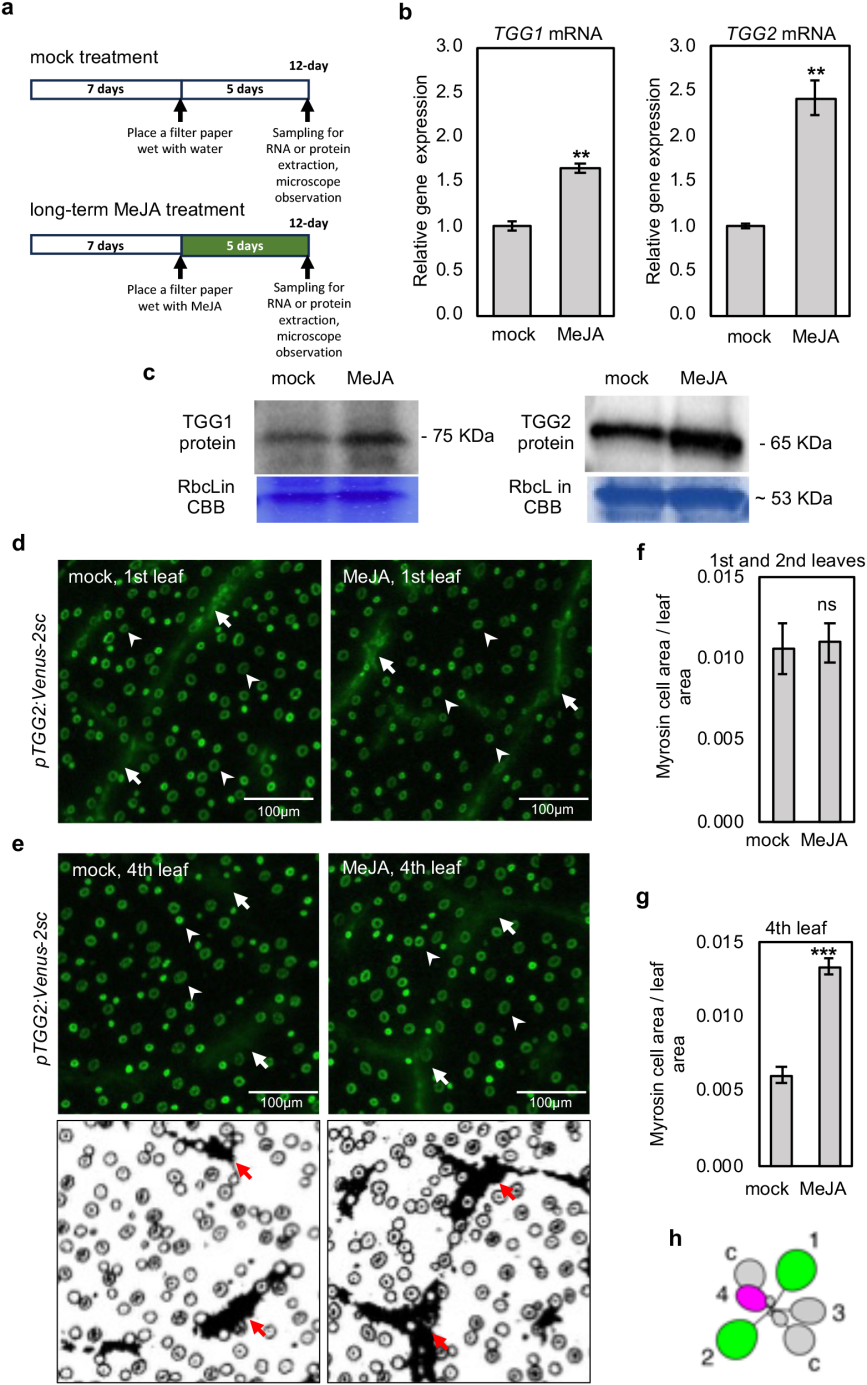
Long-term MeJA treatment increases the expression of *TGG1* and *TGG2* in wild-type rosette leaves. **(a)** The schemes show the experimental setup of mock and MeJA treatment. MeJA treatment was started 7 d after germination, and plants were sampled 12 d after germination. **(b, c)** The relative expression levels of *TGG1* and *TGG2* **(b)**, and the protein amount of TGG1 and TGG2 **(c)** in the shoots of mock or long-term MeJA treated Arabidopsis wild type (Col-0) plants. Error bars denote the standard error of three biological replications. Double asterisks denote *p* < 0.01 based on the student’s *t*-test. **(d, e)** Confocal microscopic images of the first **(d)** and fourth **(e)** leaves of 12-d-old transgenic plants harboring *pTGG2:Venus-2sc*. Myrosin cells (arrows) and stomata guard cells (arrowheads) are recognized with Venus fluorescence but have different shapes. The processed images show myrosin cells as a black area (e, lower, red arrows). **(f, g)** The ratio of myrosin cell areas per leaf area in the first and second **(f)** or fourth **(g)** leaves, which is calculated from seven independent images (e, lower). Error bars denote the standard error of seven biological replications. *** denotes *p* < 0.001 and ns denotes no significance based on the student’s *t*-test. **(h)** The leaf numbering. c, cotyledons.

The *Arabidopsis thaliana* Col-0 accession was used as the wild-type. Transgenic plants containing *ProTGG2:VENUS-2sc* and *pFAMA: GFP* constructs were kind gifts from M. Shirakawa and I. Hara-Nishimura (Shirakawa et al., 2014; Shirakawa and Hara-Nishimura, 2018). The *myc2,3,4* mutant was kindly provided by H. Frerigmann, and the *coi1–16* mutant in Col-5/Col(*gl1*) background was purchased from the Arabidopsis Biological Resource Center (ABRC). The *myc2,3,4* mutant was generated by crossing T-DNA insertion mutants *myc4* (GK491E10), *myc3* (GK445B11), and *myc2*/*jin1-9* (SALK_017005) (Frerigmann et al., 2014). The *coi1–16* mutant has a point mutation in the *COI1* gene, resulting in an amino acid alteration of L245F (Ellis and Turner, 2002). Following sterilization with 70% (v/v) ethanol, seeds were cultivated at 4 cm from the center of the plate (**Supplementary Figure 2**) and incubated at 4 °C for 2 d prior to germination. Seeds were then germinated at 22 °C under continuous light (approximately 100 µE s^-1^ m^-2^) on medium composed of 1/2 MS basal salt mixture (092623020, MP Biomedicals), 1% (w/v) sucrose and 0.5% (w/v) MES-KOH (pH 5.7) containing 0.4% (w/v) Gellan Gum (Wako, Japan). Leaves were numbered according to ontogenetic development, starting with the first true leaf, and analyzed (**Figure 1h**).

### 2.2 MeJA treatment

The liquid of MeJA (500 nmol/plate) (Sigma-Aldrich) was applied to a filter paper pad left in the cap of an Eppendorf tube (1.5 mL) for evaporation and positioned in the center of a plate (*φ* = 9 cm) (**Supplementary Figure 2**). The method was used in a previous study to examine Arabidopsis response to long-term MeJA treatment (Yoshida et al., 2009). The evaporated MeJA gradually and uniformly diffused into the enclosed air space of the plate with a saturation concentration of around 1.6 µg/L (7.13 nM in air) (Acevedo et al, 2003). This concentration was lower than the standard dose commonly used in foliar sprays (e.g., 0-100 µM, as in Feng et al., 2021). The distance between the plants and the source of MeJA was important, and the arrangement ensured uniform and equal exposure of all seedlings to MeJA. Distilled water was used instead of MeJA for the mock treatment. Leaf samples for each replicate were randomly collected, frozen in liquid nitrogen, and stored at –80 °C for subsequent molecular analyses.

### 2.3 Binocular and confocal microscope

A binocular fluorescence microscope (SteREO V12, Carl Zeiss, Jena, Germany) was used to observe fluorescent proteins in whole leaves. The images were captured using a CCD camera (AxioCam MRc5, Carl Zeiss). A confocal laser scanning microscope (LSM880, Carl Zeiss) was used to observe fluorescent proteins. We observed several leaves across different seedlings on separate plates to assess reproducibility. We used at least 5 images to calculate myrosin cell areas, fluorescence intensity, and stomata density with ImageJ.

### 2.4 RNA isolation and quantitative RT-PCR

Total RNA was extracted from the aerial parts (aboveground) of 12-d-old seedlings using the TRI Reagent (Molecular Research Center, Cincinnati, USA). After dissolving the RNA in distilled water and digesting contaminated genomic DNA with DNase I (Sigma-Aldrich), the first cDNA strand was generated from 1 µg of the RNA using Ready-to-Go RT-PCR beads (GE Healthcare) and random oligomers. PowerUp SYBR Green Master Mix (Thermo Fisher Scientific, USA) was used for the quantification of cDNA of *UBIQUITIN 10* (*UBQ10*), *TGG1*, *TGG2*, *FAMA*, *VSP2*, *BGLU18*, *JR1*, and *JAZ2* using a real time PCR thermocycler (QuantStudio 12K Flex, Thermo Fisher Scientific). All experiments were performed in three biological replications; one biological replicate is defined as three seedlings per sample. One biological replicate was analyzed with three technical replicates for qPCR. Gene-specific primer sets were generated using the Primer3Plus software (**Supplementary Table 1**). The *UBQ10* gene was used as a reference gene (Czechowski et al., 2005), because the expression levels were stable in our experiment; the PCR-cycle time (Ct) value for the control group was 24.59 ± 0.19 (standard error, *n* = 3), while the long-term MeJA-treated group was 24.16 ± 0.32, *p* = 0.27 in the Student’s t-test. Relative expression of the target genes was normalized to that of *UBQ10*. The relative expression levels were calculated using 2^-ΔΔCt^ method (Livak and Schmittgen, 2001).

### 2.5 SDS-PAGE and immunoblot analysis

Total proteins were extracted from 50 mg of leaves with 200 µL of 2× sample buffer (20 mM Tris-HCl buffer, pH 6.8, 40% (v/v) glycerol, 2% (w/v) sodium dodecyl sulfate (SDS), and 2% (v/v) 2-mercaptoethanol). The homogenate was centrifuged at 12,000 × *g* for 5 min to remove debris. Extracts (10 µL) were electrophoresed on SDS-polyacrylamide gels. After separation, the proteins were transferred onto a nylon membrane and subjected to immunoblotting. The anti-TGG1 and anti-TGG2 antibodies were diluted 5000-fold for each treatment (Ueda et al., 2006; Shirakawa et al., 2014). The proteins were stained with Coomassie Brilliant Blue R-250. The entire experiment was repeated three times to test the reproducibility.

### 2.6 Insect feeding assays

Tropical house crickets (*Gryllodes sigillatus*) of the same age (3-w-old), originating from the same colony, were purchased from a pet store in Krakow. The crickets were starved for 2 d before the experiments. The 7-d-old Arabidopsis wild type plants, *coi1–16* and *myc2,3,4* mutants were treated with either mock or MeJA for 5 d. Afterward, ten MeJA-treated and ten untreated plants were carefully removed from their growing medium and placed in a 20×20×20 cm box for the dual-choice feeding assay. To prevent desiccation of plants, their roots were wrapped in wet tissue paper. Ten starved crickets were released into each box and allowed to consume the plants by keeping them overnight. Plant weights were recorded before and after the feeding experiments. Feeding damage was expressed as percentage weight reduction to normalize for inherent growth differences between wild-type, *coi1-16*, and *myc2,3,4* plants. This approach, commonly used in insect feeding assays, allows direct comparison of susceptibility across genotypes by accounting for phenotypic size effects.

### 2.7 Accession numbers

Sequence data from this study can be obtained in the Arabidopsis Genome Initiative databases under the following accession numbers: *TGG1* (*At5g26000*), *TGG2* (*At5g25980*), *FAMA* (*At3g24140*), *COI1* (*At2g39940*), *MYC2* (*At1g32640*), *MYC3* (*At5g46760*), *MYC4* (*At4g17880*), *VSP2* (*At5g24770*), *BGLU18* (*At1g52400*), *JR1* (*At3g16470*), *JAZ2* (*At1g74950*), and *UBQ10* (*At4g05320*).

## 3 Results

### 3.1 Long-term exposure to airborne MeJA increases the expression level of *TGG1* and *TGG2* in Arabidopsis rosette leaves

JA treatment inhibits true leaf and cotyledon growth (Zhang and Turner, 2008), elongation (Chen et al., 2013), and adventitious root development (Gutierrez et al., 2012). We treated 7-d-old Arabidopsis seedlings with vaporized MeJA for 5 d (**Figure 1a**) and as previously reported (Zhang and Turner, 2008; Chen et al., 2013; Gutierrez et al., 2012), we also observed that airborne MeJA treatment reduced plant size (**Supplementary Figure 2a**). This result validated that our long-term airborne MeJA treatment system effectively induced canonical jasmonate-responsive growth inhibition. Next, we examined the expression levels of two myrosinase genes, *TGG1* and *TGG2*, after airborne MeJA treatment to determine whether JA induces the expression of these genes. Reverse transcription-quantitative PCR (RT-qPCR) analysis of shoots revealed that the expression levels of both genes were significantly increased after 5 d of airborne MeJA treatment (**Figure 1b**). In addition, immunoblot analysis showed that exposure to airborne MeJA for 5 d significantly increased TGG1 and TGG2 protein levels in the shoots (**Figure 1c**).

To trace the differentiation of myrosin cells in rosette leaves, we used a transgenic reporter line containing the *TGG2* promoter and *Venus-2sc* reporter construct (*pTGG2:Venus-2sc*), in which Venus fluorescence was observed mainly in myrosin cells and to some extent in the stomata guard cells (**Figure 1d**) (Shirakawa et al., 2016). We measured the myrosin cell area in the images based on Venus fluorescence. We divided the leaves into two types based on the leaf developmental stage at the starting point of MeJA treatment: 1) old (first and second) leaves that have completed leaf development, and 2) young (fourth) leaves that are in the course of leaf development. The myrosin cell area was not dramatically changed by MeJA treatment in the first and second leaves, but was significantly increased in the fourth leaves of MeJA-treated plants (**Figures 1e–g**). The Venus fluorescence intensity in myrosin cells did not change significantly (**Supplementary Figure 3**). We also measured the myrosin cell area of the eleventh leaves after very long-term MeJA treatment. The myrosin cell area of these young leaves tended to increase after a very long MeJA treatment (**Supplementary Figure 4**). These findings suggest that MeJA promoted myrosin cell area expansion in young leaves at certain plant ages or leaf orders. The increase in myrosin cell area can be attributed to either an increase in the number of myrosin cells or an increase in the volume of myrosin cells. Because we could not count myrosin cells in the fourth leaves, we used the emerging first leaves of younger seedlings to observe the changes in myrosin cell number after MeJA treatment. We started MeJA treatment on an earlier day (2 d after germination) when the first leaf was emerging. Microscopic observations demonstrated that the myrosin cell number was not increased by MeJA treatment in the emerging first leaf (**Supplementary Figure 5**). These findings suggest that MeJA treatment increases myrosin cell area by presumably increasing each myrosin cell volume but not by promoting myrosin cell proliferation.

As TGG2 is known to accumulate in stomatal guard cells, we counted the number of stomatal cells in MeJA-treated leaves. No significant changes in stomatal density were observed in the first, second, and fourth leaves (**Supplementary Figure 6**).

### 3.2 Long-term airborne MeJA treatment increases *TGG1* and *TGG2* expression in the canonical JA-insensitive *coi1–16* and *myc2,3,4* mutants

**Figure 2:**
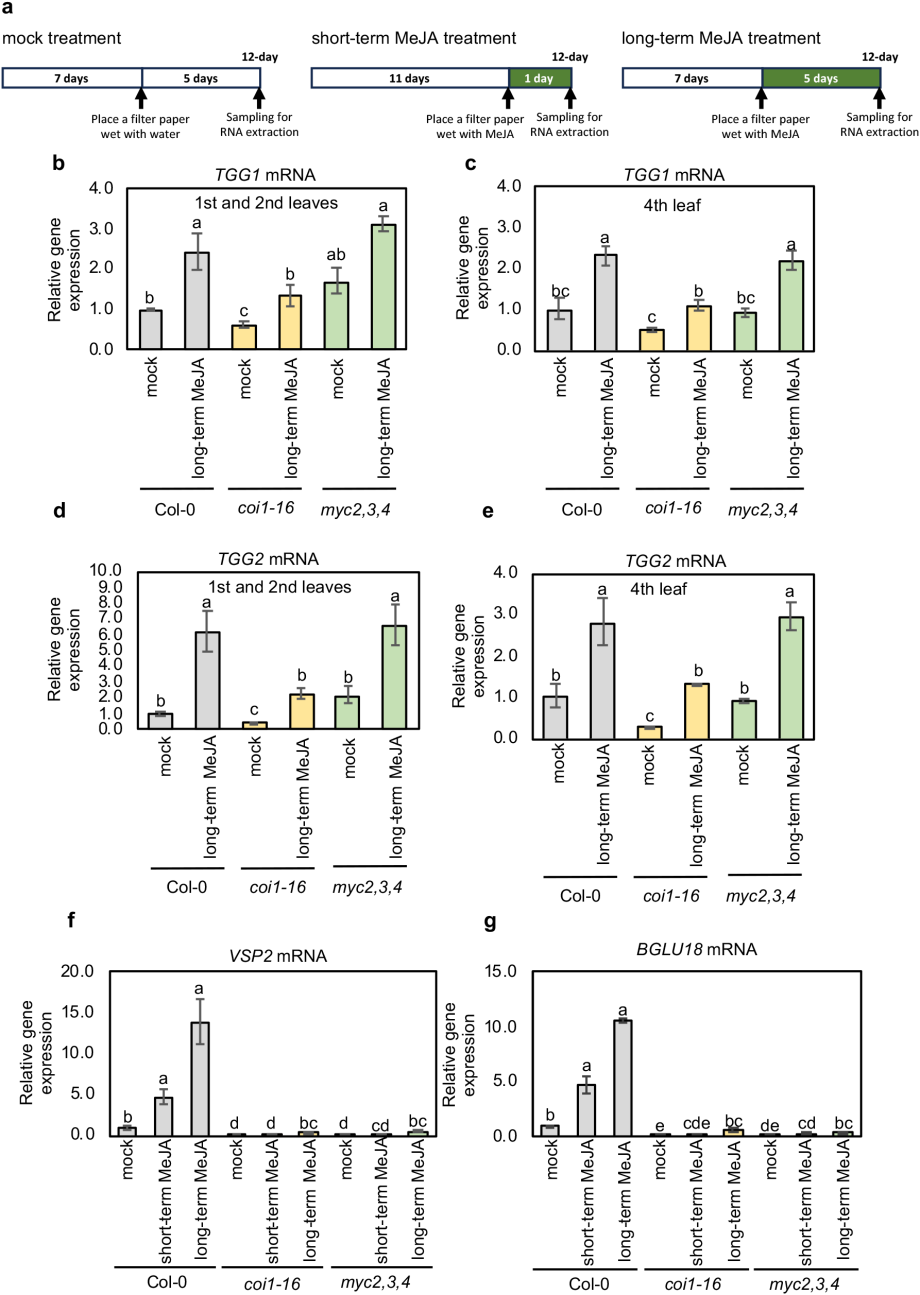
Long-term MeJA treatment increased the expression of *TGG1* and *TGG2* in the first, second, and fourth leaves of Col-0, *coi1–16* and *myc2,3,4* mutants, while the expression of canonical MeJA-responsive genes decreased significantly in *coi1–16* and *myc2,3,4* mutants. **(a)** The schemes show the experimental setup of mock, short-term and long-term MeJA treatments. MeJA treatment was started 7 (for the long-term MeJA treatment) or 11 d (for the short-term MeJA treatment) after germination, and plants were sampled 12 d after germination. **(b–e)** The relative expression levels of *TGG1* **(b, c)** and *TGG2* **(d, e)** in the first and second **(b, d)**, and fourth **(c, e)** leaves of mock and long-term MeJA treated Arabidopsis wild type (Col-0), *coi1–16* and *myc2,3,4* mutants. **(f, g)** The relative expression levels of *VSP2* **(f)** and *BGLU18* **(g)** in the shoots of mock, short- and long-term MeJA-treated Arabidopsis wild type (Col-0), *coi1–16* and *myc2,3,4* mutants. Error bars denote the standard error of three biological replications. Different lowercase letters indicate significant differences (p < 0.05; Tukey’s test).

**Figure 3:**
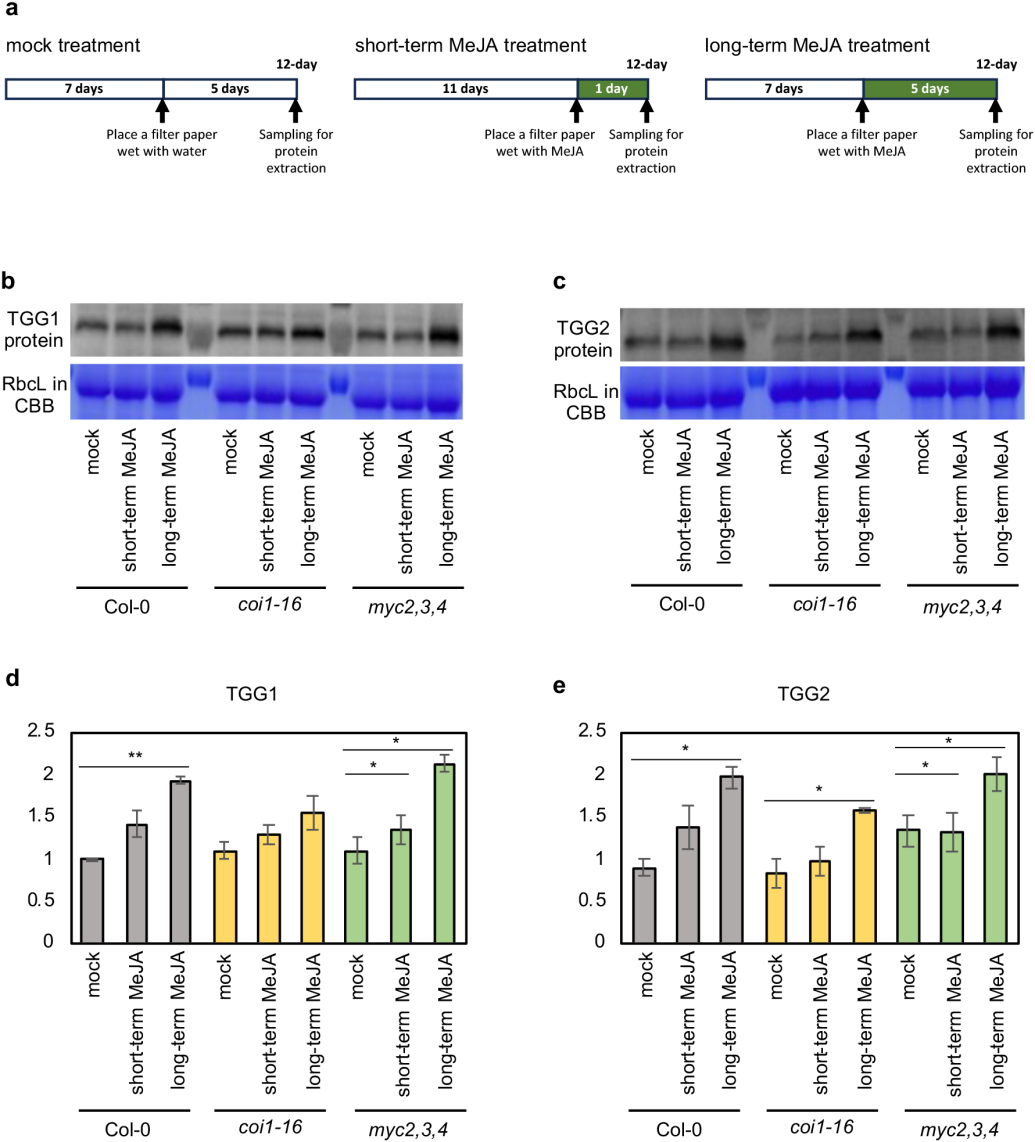
Long-term MeJA treatment increases the protein amount of TGG1 and TGG2 in *coi1–16* and *myc2,3,4* mutants. **(a)** The schemes show the experimental setup of mock, short-term and long-term MeJA treatments. MeJA treatment was started 7 (for the long-term MeJA treatment) or 11 d (for the short-term MeJA treatment) after germination, and plants were sampled 12 d after germination. **(b, c)** The protein amount of TGG1 **(b)** and TGG2 **(c)** in the shoots of mock, short- and long-term MeJA treated Arabidopsis wild type (Col-0), *coi1–16* and *myc2,3,4* mutants. **(d, e)** Bar graphs shows the densitometry of western blotting bands. Error bars indicate SE (n = 3 replicates). * and ** denotes *p* < 0.05 and *p* < 0.01 based on Tukey’s test.

COI1 is the JA receptor that forms a co-receptor complex with JAZ proteins, and MYC2, MYC3, and MYC4 are key regulators that control the expression of JA response genes (Sheard et al., 2010; Fernández-Calvo et al., 2011). To examine whether the canonical JA-signaling pathway components regulate the expression of *TGG1* and *TGG2*, we examined their expression levels in Arabidopsis *coi1* and *myc2,3,4* mutants. We used the *coi1–16* mutant allele because it is fertile at 16 °C, unlike other *coi1* mutant alleles, and can be maintained as a pure homozygous line (Ellis and Turner, 2002). Long-term (5 d) airborne MeJA treatment led to a significant increase in *TGG1* and *TGG2* expression levels in the wild-type plants. Interestingly, this response was also observed in *coi1–16* and *myc2,3,4* mutants (**Figure 2**). We examined TGG1 and TGG2 protein levels by immunoblot analysis, and the results showed that short-term (1 d) MeJA treatment did not increase TGG1 and TGG2 accumulation, but long-term treatment increased these protein levels in wild type, *coi1–16* and *myc2,3,4* plants (**Figure 3**; **Supplementary Figure 14**). Taken together, our results suggest that long-term MeJA treatment can induce *TGG* genes independently of the canonical JA signaling pathway, including COI1 and MYC2/3/4.

Recently, Feng et al. (2021) reported that 6 h MeJA treatment upregulates the *TGG1* expression in a COI1-dependent manner. To examine how long treatment is required for the stimulation of COI1-independent *TGG1* and *TGG2* expression with airborne MeJA treatment, we conducted a time-course experiment by changing the starting date for the MeJA treatment in wild-type and *coi1–16* plants. The result revealed that both *TGG1* and *TGG2* expressions were significantly increased after 2 d MeJA treatment in wild type, and after 4 d for *TGG1* and 2 d for *TGG2* in *coi1–16* mutant (**Supplementary Figure 7**).

As the *coi1–16* and *myc2,3,4* mutants have been described to have a leaky JA response (Ellis and Turner, 2002; Song et al., 2017), and the extent of the JA response in these mutants was not known in the long-term airborne MeJA treatment, we examined the plant growth phenotype of these mutants in the long-term airborne MeJA treatment under the same conditions in **Figure 1a**. Airborne MeJA treatment reduced shoot and root growth and shoot fresh weight (**Supplementary Figure 8**) in the wild type, but not in the *coi1–16* and *myc2,3,4* mutants. These results indicate that *coi1–16* and *myc2,3,4* mutants reduced or eliminated the MeJA response under our experimental conditions.

To further investigate the response of *coi1–16* and *myc2,3,4* mutants to airborne MeJA treatment, we examined the expression levels of the known COI1-dependent JA-inducible genes *VSP2*, *BGLU18*, *JR1*, and *JAZ2* (Boter et al., 2004; Chung et al., 2009). The expression levels of *VSP2* and *BGLU18* were significantly increased after short-term (1 d) airborne MeJA treatment, and these levels were further increased by long-term (5 d) airborne MeJA treatment in wild-type plants. In contrast, *coi1–16* and *myc2,3,4* mutant seedlings showed strongly reduced expression of *VSP2* and *BGLU18* under the same treatment (**Figures 2f, g**). Similar results were observed for *JR1* and *JAZ2*, and their expressions were significantly lower in *coi1–16* and *myc2,3,4* mutants than in wild-type plants after long-term MeJA treatment (**Supplementary Figure 9**). These results indicate that *VSP2*, *BGLU18*, *JR1*, and *JAZ2* expressions are dependent on COI1 and MYC2/3/4, suggesting that the pathway regulating *TGG1* and *TGG2* expression is distinct from that regulating the *VSP2*, *BGLU18*, *JR1*, and *JAZ2* under long-term MeJA treatment.

### 3.3 Long-term MeJA treatment increased *FAMA* expression

**Figure 4:**
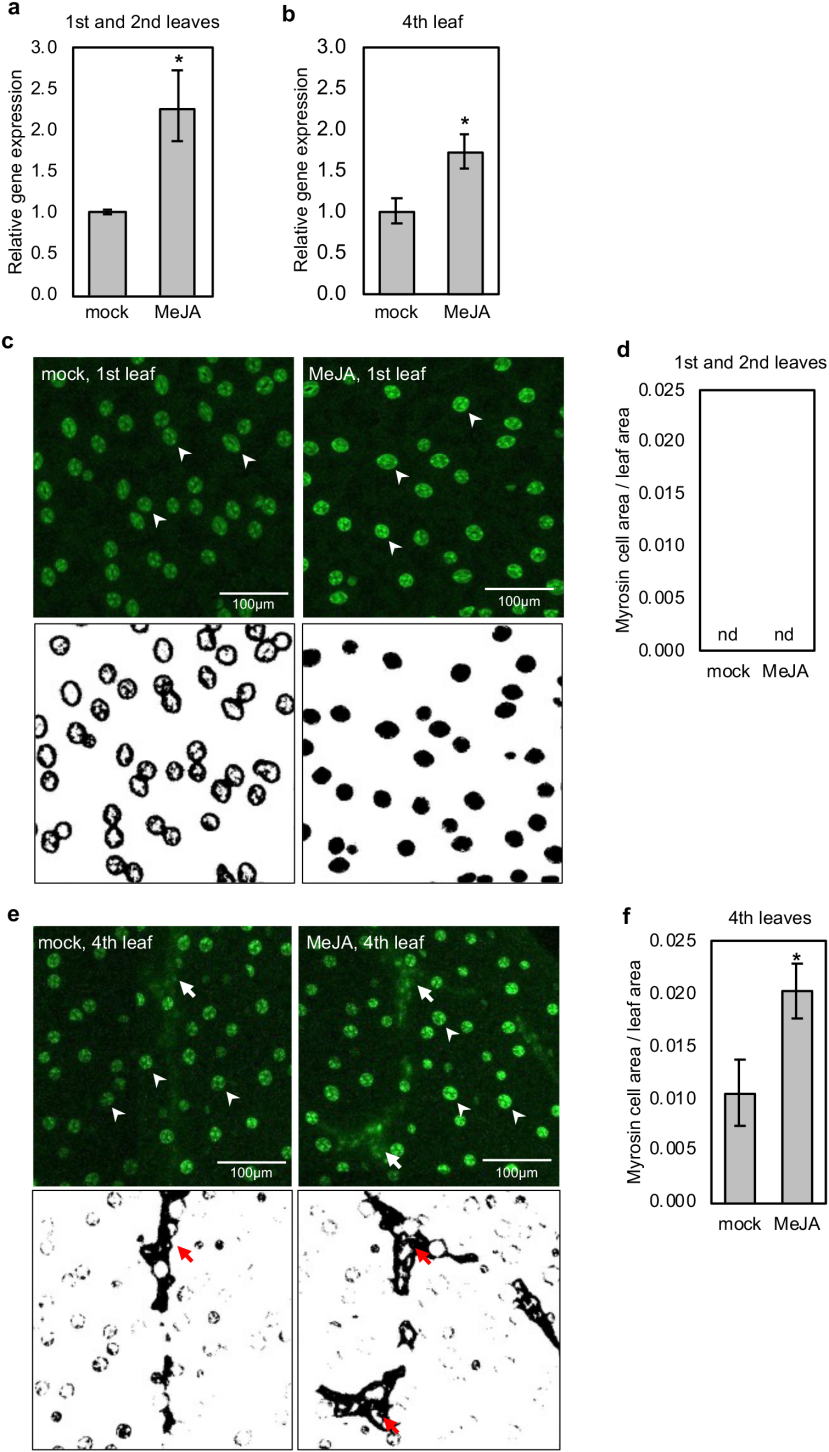
Long-term MeJA treatment increased the expression of *FAMA* in the first, second, and fourth leaves. **(a, b)** The relative expression levels of *FAMA* in different rosette leaves of 12-d-old Arabidopsis wild-type (Col-0) plants. Error bars denote the standard error of three biological replications. * denotes *p* < 0.05 based on the student’s *t*-test. **(c, e)** Confocal microscopic images (upper) of the first **(c)** and fourth **(e)** leaves of 12-d-old transgenic plants harboring *pFAMA: GFP*. Arrows and arrowheads represent myrosin cells and stomata guard cells, respectively. The processed images (lower) show myrosin cells as a black area (red arrows). **(d, f)** The ratio of GFP-expressing myrosin cell areas per leaf area in the first and second **(d)** or fourth **(f)** leaves, which is calculated from five independent images (c, e, lower). Error bars denote the standard error of five biological replications. * denotes *p* < 0.05 based on the student’s *t*-test.

Because FAMA is a regulator of myrosin cell differentiation and *TGGs* expression (Li and Sack, 2014; Shirakawa et al., 2014; Feng et al., 2021), we further investigated the possible role of MeJA in increasing myrosin cell differentiation by monitoring *FAMA* expression. Long-term MeJA treatment upregulated the expression of *FAMA* in the first, second, and fourth leaves of both wild-type and *coi1–16* mutant plants, while no significant change in *FAMA* expression was observed in *myc2,3,4* mutant (**Figures 4a, b**; **Supplementary Figure 10**). *FAMA* is expressed in both stomatal guard and myrosin cells. To examine the *FAMA* expression patterns in stomatal guard cells and myrosin cells, we used *pFAMA: GFP* transgenic plants (Shirakawa and Hara-Nishimura, 2018). Long-term MeJA treatment increased the GFP fluorescence intensity of stomatal guard cells in the first, second, and fourth leaves, indicating an increase in *FAMA* expression in these cells (**Supplementary Figure 11**). Regarding the myrosin cells area, a significant increase was observed in the fourth leaves of 12-d-old MeJA-treated plants compared to mock plants, but no *FAMA* expression was observed in the myrosin cells of the first and second leaves regardless of MeJA treatment (**Figures 4c–f**). These findings suggest that the increase in *FAMA* expression in the first and second leaves of MeJA-treated plants is attributable to its increased expression in stomatal cells, whereas the increase in *FAMA* expression in the fourth leaf is a consequence of its higher expression in stomatal and myrosin cells.

### 3.4 Long-term MeJA treatment decreased the feeding preference of crickets

**Figure 5:**
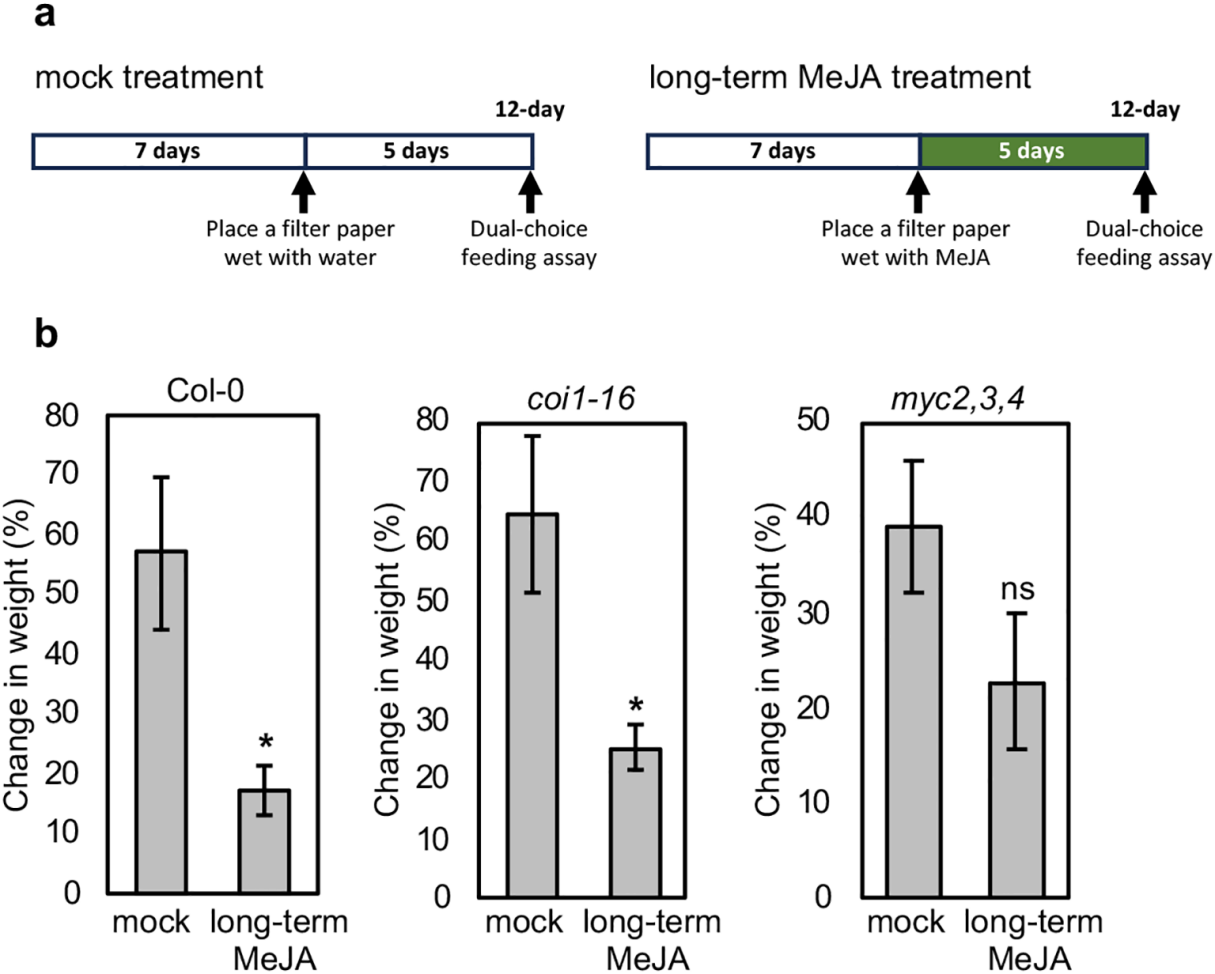
Effect of long-term MeJA treatment on cricket feeding preference. **(a)** The schemes show the experimental setup of mock and long-term MeJA treatments. MeJA treatment was started 7 d after germination, and 12-d-old plants were subjected to the dual-choice feeding assay. **(b)** The chart shows the ratio of plant weight reduction by feeding. Crickets of the same age, originating from the same colony, were starved for 2 d before the experiments. The 7-d-old Arabidopsis wild type plants, *coi1–16* and *myc2,3,4* mutants, were treated with either mock or MeJA for 5 d. Afterward, ten MeJA-treated and untreated plants were carefully transferred from their growing medium into a 20×20×20 cm box (a dual-choice feeding assay). Ten starved crickets were released into each box and kept overnight. Plant weights were recorded before and after the feeding experiments and calculated as a change in weight (%). Error bars indicate standard error of four independent experiments (*n* = 4). Significance values were calculated by a two-sided Student’s *t*-test. Asterisk denotes *p* < 0.05, and ns denotes no significance based on the Student’s *t*-test.

To examine the effect of long-term MeJA treatment on defense against herbivores, we performed dual-choice feeding assays using crickets (*Gryllodes sigillatus*). The 7-d-old wild-type plants and *coi1–16* and *myc2,3,4* mutants were either mock- or MeJA-treated for 5 d before being exposed to starved crickets. In wild-type plants, long-term MeJA treatment reduced cricket feeding compared to that of mock-treated plants (**Figure 5**; **Supplementary Figure 12**), indicating that long-term MeJA treatment enhanced plant defense against herbivores. In addition, surprisingly, the *coi1–16* significantly increased defense against herbivores following long-term MeJA treatment, and this trend was also observed in the *myc2,3,4* mutant (**Figure 5**; **Supplementary Figure 12**), suggesting that long-term MeJA treatment enhanced herbivory resistance in these mutants which are impaired in canonical JA signaling.

## 4 Discussion

In nature, plants are constantly subjected to stresses such as wounding or herbivore attacks, which induce JA biosynthesis and subsequently activate the JA signaling pathway (McConn et al., 1997; Halitschke and Baldwin, 2004). Exposure of plants to exogenous MeJA elicits stress responses by activating JA-dependent signaling pathways (Jiang et al., 2017). In this study, we found that airborne MeJA signals induce defense-related myrosinase genes, namely *TGG1* and *TGG2*, in Arabidopsis plants. However, we unexpectedly found that long-term MeJA treatment induced the expression of *TGG1* and *TGG2* independent of the canonical JA signaling pathway using COI1 and MYC2/3/4.

It has been previously reported that a 6 h MeJA treatment induces the expression of *TGG1* but reduces the expression of *TGG2* (Feng et al., 2021). Under our experimental conditions, we did not observe any significant changes in the expression of *TGG1* until 2 d of MeJA treatment. This discrepancy may be due to the method of MeJA treatment; in this study slowly evaporating MeJA was applied to the plants through the air inside the plates, whereas MeJA was directly applied to the plants in the study by Feng et al. (2021). In our time course experiment, the upregulation of *TGG1* and *TGG2* in wild type started after 2 d of MeJA treatment, whereas the increase of *TGG1* and *TGG2* in *coi1–16* started after 4 d and 2 d of MeJA treatment, respectively. These findings suggest that the COI1-independent induction is activated after 2 d of MeJA treatment; however, the response of *TGG1* to the COI1-independent induction is slower than that of *TGG2*.

Long-term airborne MeJA treatment induces *TGG1* and *TGG2*, but not entirely through COI1 and MYC2/3/4 signaling pathways. Under long-term MeJA treatment, *TGG1* and *TGG2* gene expression increased in both *coi1–16* and *myc2,3,4* mutants; however, typical JA responses, such as *VSP2* and *BGLU18* gene expression (Liu and Timko, 2021; Schweizer et al., 2013; Stefanik et al., 2020) and the induction of growth inhibition (Zhang and Turner, 2008), were attenuated in the mutants compared to the wild type. Therefore, the regulatory system of *TGG1* and *TGG2* in response to long-term MeJA treatment is a newly identified process that is poorly understood.

MYC2/3/4 are TFs involved in canonical JA signaling; however, our analyses indicated that these TFs are not involved in the regulation of *TGG1* and *TGG2* expression in response to long-term MeJA treatment. Despite their well-established role in short-term JA responses, mutations in *myc2/3/4* did not prevent the *TGG1* and *TGG2* induction, suggesting that prolonged exposure to MeJA activates regulatory mechanisms that operate independently of the canonical MYC module. It is possible that the residual canonical JA signaling pathway or the indirect effect of JA promotes *TGG1* and *TGG2* expression. For example, a MYC2/3/4 homologue, namely MYC5 (Figueroa and Browse, 2015; Song et al., 2017), may act redundantly to induce *TGG1* and *TGG2* expression in the *myc2,3,4* mutants. Alternatively, this finding may challenge the prevailing JA signaling model and support the existence of an alternative transcriptional control pathway that is activated during sustained stress. FAMA is a master TF for myrosin and stomatal guard cell differentiation, and *TGG1* and *TGG2* expression (Li and Sack, 2014; Shirakawa et al., 2014; Feng et al., 2021). In our experiment, long-term MeJA treatment led to a pronounced increase in *FAMA* expression in the first, second, and fourth rosette leaves, suggesting that FAMA is the TF for the expression of *TGG1* and *TGG2* after long-term MeJA treatment. Due to the pleiotropic growth defect in *fama* mutants (Ohashi-Ito and Bergmann, 2006), we could not assess their response to long-term JA treatment. Nevertheless, our results are consistent with previous observations that an increase in *FAMA* transcripts correlates with the activation of *TGG1* and *TGG2* expression (Feng et al., 2021). These data suggest that *FAMA* expression is a hallmark of sustained activation of downstream *TGG1* and *TGG2* gene expression, although further evaluation is required to determine whether FAMA is involved in the activation of these genes during long-term MeJA treatment.

Our microscopic observation demonstrated consistent *FAMA* expression in stomatal guard cells. Long-term MeJA treatment increased *FAMA* expression in stomatal guard cells but not in myrosin cells in older, earlier emerged leaves, suggesting that the increased expression of *TGGs* in older, earlier-emerged leaves likely comes from stomatal cells. In contrast, in 12-d-old plants, the expression of *FAMA* was absent in myrosin cells in the first and second leaves but detectable in the fourth leaf, pointing to developmental or positional differences in JA responsiveness. Together, these observations support a model in which the JA signaling outputs vary depending on leaf age or phyllotaxy, with younger, later-emerged leaves responding to prolonged JA treatment in both stomatal guard cells and myrosin cells, whereas older, early-emerged leaves show a more restricted stomatal guard cells-specific response (**Supplementary Figure 13**).

In addition to transcriptional regulation, long-term MeJA treatment had a positive effect on the myrosin cell area in young leaves at certain developmental stages, indicating that this phenomenon was positively correlated with the upregulation of *TGG1* and *TGG2* expression in shoots. Notably, long-term MeJA treatment did not increase the number of myrosin cells (**Supplementary Figure 5**), suggesting that MeJA does not promote myrosin cell differentiation. Therefore, it can be concluded that the long-term exposure to MeJA enhances myrosin cell expansion, along with *FAMA*, *TGG1*, and *TGG2* expressions at certain developmental stages or leaf orders. This interpretation is supported by previous findings showing that MeJA can inhibit cell cycle progression at the G1 phase, leading to increased cell size in both leaf and root tissues (Noir et al., 2013). Thus, prolonged MeJA signaling may coordinately regulate transcriptional activation and cellular growth to reinforce myrosinase-based defense capacity.

There are examples of COI1-independent JA responses in plants. JA inhibits the formation of lateral roots through the auxin action, and its inhibitory effect is still observed in Arabidopsis *coi1* mutant, indicating that the effect is COI1-independent (Ishimaru et al., 2018). In this phenomenon, JA prevents the degradation of AUXIN (Aux)/IAA repressor proteins. This JA activity on lateral root inhibition was reduced in *auxin signaling f-box protein 5* (*afb5*) mutant, suggesting that the AFB5 protein is involved in stabilizing Aux/IAA proteins in the COI1-independent JA signaling pathway (Ishimaru et al., 2018). In another study, a COI1-independent signaling pathway has been reported in which exogenous JA-Ile treatment induces a rapid cytosolic calcium (Ca^2+^) increase in Arabidopsis leaf cells. The Arabidopsis double knockout mutant of *plant elicitor peptide receptor 1* (*perp1*) and *perp2* reduced rapid cytosolic Ca^2+^ increase and subsequent JA-Ile responsive genes against exogenous JA-Ile treatment, suggesting that PERP1 and PERP2 are involved in a COI1-independent JA-Ile-signaling pathway (Mittal et al., 2024). Integrating these studies with our results suggests that while the COI1-dependent pathway is the main pathway for JA perception, COI1-independent mechanisms regulate a distinct subset of JA-responsive genes.

Our conclusions regarding the COI1- and MYC-independent contribution of *TGG1* and *TGG2* to long-term MeJA-induced defense are further supported by insect feeding assay. The consistent feeding resistance observed in this assay strongly supports the idea that long-term MeJA exposure engages defense pathways that are at least partially independent of canonical COI1 signaling pathway. Further analysis employing other JA related mutants or metabolomic analysis will be suited to dissect the canonical and non-canonical JA signaling pathway, and tier contributions on the TGG1 and TGG2 mediated defense. Nevertheless, our presented data strongly indicate that long-term MeJA responses engage alternative or compensatory regulatory pathways.

It was assumed that MeJA signaling relies mainly on COI1. However, our findings show that TGG1 and TGG2 expression can also be induced in a partially COI1-independent manner. This suggests that plants might evolve an alternative pathway as a backup to bypass COI1 canonical pathway. It is likely that additional COI1-independent genes will be identified, further clarifying the precise regulatory mechanisms that governs this alternative signaling pathway. This study underscores the importance of treatment duration and experimental context in JA signaling studies. Collectively, our data shift our perspective on JA signaling and reveals how versatile a plant’s chemical defense toolkit can be.

## Data Availability

The raw data supporting the conclusions of this article will be made available by the authors, without undue reservation.

## References

[B1] Acevedo C. Sanchez E. Young M. E. Simpson R. (2003). Prediction correlation of vapor pressure for methyl jasmonate. J. Food Eng. 59, 431–433. doi: 10.1016/S0260-8774(03)00004-9, PMID: 41810140

[B2] Andersson D. Chakrabarty R. Bejai S. Zhang J. Rask L. Meijer J. (2009). Myrosinases from root and leaves of *Arabidopsis thaliana* have different catalytic properties. Phytochemistry 70, 1345–1354. doi: 10.1016/j.phytochem.2009.07.036, PMID: 19703694

[B3] Bhat R. Vyas D. (2019). Myrosinase: insights on structural, catalytic, regulatory, and environmental interactions. Crit. Rev. Biotechnol. 39, 508–523. doi: 10.1080/07388551.2019.1576024, PMID: 30939944

[B4] Bones A. M. Rossiter J. T. (1996). The myrosinase-glucosinolate system, its organisation and biochemistry. Physiol. Plant. 97, 194–208. doi: 10.1111/j.1399-3054.1996.tb00497.x, PMID: 41875165

[B5] Boter M. Ruíz-Rivero O. Abdeen A. Prat S. (2004). Conserved MYC transcription factors play a key role in jasmonate signaling both in tomato and Arabidopsis. Genes Dev. 18, 1577–1591. doi: 10.1101/gad.297704, PMID: 15231736 PMC443520

[B6] Bussy A. (1840). Sur la formation de l’huile essentielle de Moutarde. J. Pharm. 27, 464–471.

[B7] Capella A. Menossi M. Arruda P. Benedetti C. (2001). COI1 affects myrosinase activity and controls the expression of two flower-specific myrosinase-binding protein homologues in *Arabidopsis*. Planta 213, 691–699. doi: 10.1007/s004250100548, PMID: 11678272

[B8] Chen J. Sonobe K. Ogawa N. Masuda S. Nagatani A. Kobayashi Y. . (2013). Inhibition of *Arabidopsis* hypocotyl elongation by jasmonates is enhanced under red light in phytochrome B dependent manner. J. Plant Res. 126, 161–168. doi: 10.1007/s10265-012-0509-3, PMID: 22825635 PMC3530149

[B9] Chini A. Fonseca S. Chico J. M. Fernández-Calvo P. Solano R. (2009). The ZIM domain mediates homo- and heteromeric interactions between *Arabidopsis* JAZ proteins. Plant J. 59, 77–87. doi: 10.1111/j.1365-313X.2009.03852.x, PMID: 19309455

[B10] Chung H. S. Niu Y. Browse J. Howe G. A. (2009). Top hits in contemporary JAZ: an update on jasmonate signaling. Phytochemistry 70, 1547–1559. doi: 10.1016/j.phytochem.2009.08.022, PMID: 19800644 PMC3271379

[B11] Czechowski T. Stitt M. Altmann T. Udvardi M. K. Scheible W. R. (2005). Genome-wide identification and testing of superior reference genes for transcript normalization in *Arabidopsis*. Plant Physiol. 139, 5–17. doi: 10.1104/pp.105.063743, PMID: 16166256 PMC1203353

[B12] Devoto A. Nieto-Rostro M. Xie D. Ellis C. Harmston R. Patrick E. . (2002). COI1 links jasmonate signalling and fertility to the SCF ubiquitin–ligase complex in *Arabidopsis*. Plant J. 32, 457–466. doi: 10.1046/j.1365-313X.2002.01432.x, PMID: 12445118

[B13] Ellis C. Turner J. G. (2002). A conditionally fertile coi1 allele indicates cross-talk between plant hormone signalling pathways in *Arabidopsis thaliana* seeds and young seedlings. Planta 215, 549–556. doi: 10.1007/s00425-002-0787-4, PMID: 12172836

[B14] Feng Q. Li L. Liu Y. Shao X. Li X. (2021). Jasmonate regulates the FAMA/mediator complex subunit 8-THIOGLUCOSIDE GLUCOHYDROLASE 1 cascade and myrosinase activity. Plant Physiol. 187, 963–980. doi: 10.1093/plphys/kiab283, PMID: 34608953 PMC8491074

[B15] Fernández-Calvo P. Chinim A. Fernández-Barbero G. Chico J. M. Gimenez-Ibanez S. Geerinck J. . (2011). The *Arabidopsis* bHLH transcription factors MYC3 and MYC4 are targets of JAZ repressors and act additively with MYC2 in the activation of jasmonate responses. Plant Cell 23, 701–715. doi: 10.1105/tpc.110.080788, PMID: 21335373 PMC3077776

[B16] Figueroa P. Browse J. (2015). Male sterility in Arabidopsis induced by overexpression of a MYC5-SRDX chimeric repressor. Plant J. 81, 849–860. doi: 10.1111/tpj.12776, PMID: 25627909

[B17] Frerigmann H. Berger B. Gigolashvili T. (2014). bHLH05 is an interaction partner of MYB51 and a novel regulator of glucosinolate biosynthesis in *Arabidopsis*. Plant Physiol. 166, 349–369. doi: 10.1104/pp.114.240887, PMID: 25049362 PMC4149720

[B18] Guignard L. (1890). Recherches sur la localisation des principes actifs des Crucifères. J. Bot. 4, 385–395.

[B19] Gutierrez L. Mongelard G. Floková K. Pǎcurar D. I. Novák O. Staswick P. . (2012). Auxin controls *Arabidopsis* adventitious root initiation by regulating jasmonic acid homeostasis. Plant Cell 24, 2515–2527. doi: 10.1105/tpc.112.099119, PMID: 22730403 PMC3406919

[B20] Halitschke R. Baldwin I. T. (2004). Jasmonates and related compounds in plant-insect interactions. J. Plant Growth Regul. 23, 238–245. doi: 10.1007/s00344-004-0037-z, PMID: 41878318

[B21] Heinricher E. (1884). Uber Eiweissstoffe fuhrende Idioblasten bei einigen Cruciferen. Ber. Dtsch. Bot. Ges 2, 463–467. doi: 10.1111/j.1438-8677.1884.tb07658.x, PMID: 41875165

[B22] Ishimaru Y. Hayashi K. Suzuki T. Fukaki H. Prusinska J. Meester C. . (2018). Jasmonic acid inhibits auxin-induced lateral rooting independently of the CORONATINE INSENSITIVE1 receptor. Plant Physiol. 177, 1704–1716. doi: 10.1104/pp.18.00357, PMID: 29934297 PMC6084677

[B23] Jiang Y. Ye J. Li S. Niinemets Ü. (2017). Methyl jasmonate-induced emission of biogenic volatiles is biphasic in cucumber: a high-resolution analysis of dose dependence. J. Exp. Bot. 68, 4679–4694. doi: 10.1093/jxb/erx244, PMID: 28981785 PMC5853251

[B24] Li M. Sack F. D. (2014). Myrosin idioblast cell fate and development are regulated by the *Arabidopsis* transcription factor FAMA, the auxin pathway, and vesicular trafficking. Plant Cell 26, 4053–4066. doi: 10.1105/tpc.114.129726, PMID: 25304201 PMC4247575

[B25] Liu H. Timko M. P. (2021). Jasmonic acid signaling and molecular crosstalk with other phytohormones. Int. J. Mol. Sci. 22, 2914. doi: 10.3390/ijms22062914, PMID: 33805647 PMC8000993

[B26] Livak K. J. Schmittgen T. D. (2001). Analysis of relative gene expression data using real-time quantitative PCR and the 2^-ΔΔCt^ method. Methods 25, 402–408. doi: 10.1006/meth.2001.1262, PMID: 11846609

[B27] McConn M. Creelman R. A. Bell E. Mullet J. E. Browse J. (1997). Jasmonate is essential for insect defense. Proc. Natl. Acad. Sci U.S.A. 94, 5473–5477. doi: 10.1073/pnas.94.10.5473, PMID: 11038546 PMC24703

[B28] Mittal D. Gautam J. K. Varma M. Laie A. Mishra S. Behera S. . (2024). External jasmonic acid isoleucine mediates amplification of plant elicitor peptide receptor (PEPR) and jasmonate-based immune signallin. Plant Cell Environ. 47, 1397–1415. doi: 10.1111/pce.14812, PMID: 38229005

[B29] Noir S. Bömer M. Takahashi N. Ishida T. Tsui T. L. Balbi V. . (2013). Jasmonate controls leaf growth by repressing cell proliferation and the onset of endoreduplication while maintaining a potential stand-by mode. Plant Physiol. 161, 1930–1951. doi: 10.1104/pp.113.214908, PMID: 23439917 PMC3613466

[B30] Ohashi-Ito K. Bergmann D. C. (2006). *Arabidopsis* FAMA controls the final proliferation/differentiation switch during stomatal development. Plant Cell 8, 2493–2505. doi: 10.1105/tpc.106.046136, PMID: 17088607 PMC1626605

[B31] Rask L. Andréasson E. Ekbom B. Eriksson S. Pontoppidan B. Meijer J. (2000). Myrosinase: gene family evolution and herbivore defense in Brassicaceae. Plant Mol. Biol. 42, 93–114. doi: 10.1023/A:1006380021658, PMID: 10688132

[B32] Ruan J. Zhou Y. Zhou M. Yan J. Khurshid M. Weng W. . (2019). Jasmonic acid signaling pathway in plants. Int. J. Mol. Sci. 20, 2479. doi: 10.3390/ijms20102479, PMID: 31137463 PMC6566436

[B33] Schweizer F. Fernández-Calvo P. Zander M. Diez-Diaz M. Fonseca S. Glauser G. . (2013). *Arabidopsis* basic helix-loop-helix transcription factors MYC2, MYC3, and MYC4 regulate glucosinolate biosynthesis, insect performance, and feeding behavior. Plant Cell 25, 3117–3132. doi: 10.1105/tpc.113.115139, PMID: 23943862 PMC3784603

[B34] Sheard L. B. Tan X. Mao H. Withers J. Ben-Nissan G. Hinds T. R. . (2010). Jasmonate perception by inositol-phosphate-potentiated COI1-JAZ co-receptor. Nature 468, 400–405. doi: 10.1038/nature09430, PMID: 20927106 PMC2988090

[B35] Shirakawa M. Hara-Nishimura I. (2018). Specialized vacuoles of myrosin cells: Chemical defense strategy in Brassicales plants. Plant Cell Physiol. 59, 1309–1316. doi: 10.1093/pcp/pcy082, PMID: 29897512

[B36] Shirakawa M. Tanida M. Ito T. (2022). The cell differentiation of idioblast myrosin cells: similarities with vascular and guard cells. Front. Plant Sci. 12. doi: 10.3389/fpls.2021.829541, PMID: 35082820 PMC8784778

[B37] Shirakawa M. Ueda H. Nagano A. J. Shimada T. Kohchi T. Hara-Nishimura I. (2014). FAMA is an essential component for the differentiation of two distinct cell types, myrosin cells and guard cells, in *Arabidopsis*. Plant Cell 26, 4039–4052. doi: 10.1105/tpc.114.129874, PMID: 25304202 PMC4247577

[B38] Shirakawa M. Ueda H. Shimada T. Hara-Nishimura I. (2016). Myrosin cells are differentiated directly from ground meristem cells and are developmentally independent of the vasculature in *Arabidopsis* leaves. Plant Signaling Behav. 11, e1150403. doi: 10.1080/15592324.2016.1150403, PMID: 26967973 PMC4883950

[B39] Song S. Huang H. Wang J. Liu B. Qi T. Xie D. (2017). *MYC5* is involved in jasmonate-regulated plant growth, leaf senescence and defense responses. Plant Cell Physiol. 58, 1752–1763. doi: 10.1093/pcp/pcx112, PMID: 29017003

[B40] Stefanik N. Bizan J. Wilkens A. Tarnawska-Glatt K. Goto-Yamada S. Strzałka K. . (2020). NAI2 and TSA1 drive differentiation of constitutive and inducible ER body formation in brassicaceae. Plant Cell Physiol. 61, 722–734. doi: 10.1093/pcp/pcz236, PMID: 31879762

[B41] Ueda H. Nishiyama C. Shimada T. Koumoto Y. Hayashi Y. Kondo M. . (2006). AtVAM3 is required for normal specification of idioblasts, myrosin cells. Plant Cell Physiol. 47, 164–175. doi: 10.1093/pcp/pci232, PMID: 16306062

[B42] Wang M. Sun X. Tan D. Gong S. Meijer J. Zhang J. (2009). The two non-functional myrosinase genes TGG3 and TGG6 in *Arabidopsis* are expressed predominantly in pollen. Plant Sci. 177, 371–375. doi: 10.1016/J.PLANTSCI.2009.06.003, PMID: 41881759

[B43] Xie D. X. Feys B. F. James S. Nieto-Rostro M. Turner J. G. (1998). COI1: An *Arabidopsis* gene required for jasmonate-regulated defense and fertility. Science 280, 1091–1094. doi: 10.1126/science.280.5366.1091, PMID: 9582125

[B44] Yoshida Y. Sano R. Wada T. Takabayashi. J. Okada. K. (2009). Jasmonic acid control of GRABRA3 links inducible defense and trichome patterning in *Arabidopsis*. Development 136, 1039–1048. doi: 10.1242/dev.030585, PMID: 19234066

[B45] Zhang K. Su H. Zhou J. Liang W. Liu D. Li J. (2019). Overexpressing the myrosinase gene TGG1 enhances stomatal defense against *Pseudomonas syringae* and delays flowering in Arabidopsis. Front. Plant Sci. 10. doi: 10.3389/fpls.2019.01230, PMID: 31636648 PMC6787276

[B46] Zhang Y. Turner J. G. (2008). Wound-induced endogenous jasmonates stunt plant growth by inhibiting mitosis. PLoS One 3, e3699. doi: 10.1371/journal.pone.0003699, PMID: 19002244 PMC2577035

